# Examining Care Network Characteristics in Older Adults’ Relocation to Residential Care Settings

**DOI:** 10.1093/geroni/igae087

**Published:** 2024-09-26

**Authors:** Natasha Nemmers, Wenhua Lai, Sophia Tsuker, Srabani Haldar, Vicki A Freedman, Amanda N Leggett

**Affiliations:** Institute of Gerontology, Wayne State University, Detroit, Michigan, USA; Institute of Gerontology, Wayne State University, Detroit, Michigan, USA; Institute of Gerontology, Wayne State University, Detroit, Michigan, USA; Institute of Gerontology, Wayne State University, Detroit, Michigan, USA; Institute for Social Research, University of Michigan, Ann Arbor, Michigan, USA; Institute of Gerontology, Wayne State University, Detroit, Michigan, USA

**Keywords:** Activities of daily living, Aging in place, Assisted living, Caregiving, Nursing home

## Abstract

**Background and Objectives:**

When older adults face increasing care needs or limited support, remaining safely and comfortably at home becomes challenging. Extant research has primarily concentrated on characteristics of the older adult or their primary caregiver on nursing home admission. This study examines the risk of older adults transitioning to residential care (e.g., assisted living, nursing home), focusing on the influence of their care network or involvement of multiple helpers.

**Research Design and Methods:**

Using the National Health and Aging Trends Study, we conducted competing risk regression models that account for mortality, following 7,085 initially community-dwelling older adults across Rounds 1–9 (2011–2019). We assessed network composition, size, shared tasks, and the number of in-network specialists or generalists while controlling for individual sociodemographic and health factors.

**Results:**

Individuals with care networks that shared medical tasks had the highest risk of moving to a residential care setting, followed by those sharing household tasks. Conversely, shared mobility or self-care and transportation responsibilities were associated with lower risks. Having more generalists, but not specialists, increased the risk. Larger networks were associated with heightened risk, although having close family members like a spouse was protective.

**Discussion and Implications:**

The findings underscore that care network characteristics are critical to older adults’ ability to age in place. Specifically, older adults with larger networks, lacking a spouse or child, and providing complex care are at greater risk for relocating. Understanding care networks can guide interventions related to care network coordination and resource allocation to help avoid or postpone a residential care move.

**Translational Significance:** This study examines how care network characteristics, such as size, composition, and task-sharing dynamics, affect the risk of community-dwelling older adults transitioning to residential care settings. We identify high-risk individuals, including those with large networks, without a close family member, or who rely on shared medical and household tasks across multiple helpers. The findings highlight the need to consider broader care networks beyond the older adult or a primary caregiver. These insights can guide interventions and policies to optimize network coordination, family-centered support, and resource allocation, thereby supporting aging in place and potentially delaying transitions to residential care.

Most older adults prefer to age in place, meaning they wish to remain safely, independently, and comfortably in their own homes and communities (AARP, 2021). However, several barriers, including an increased need for assistance with daily activities, impede this preference (Iecovich, 2014). Family and friends play a crucial role as a primary source of support for individuals with dementia or disabilities, enabling them to continue living in their communities (Kasper et al., 2015; Wolf et al., 2018). At the same time, there have been significant shifts toward alternative residential care options to nursing homes, such as independent and assisted living (Chyr et al., 2020; Freedman & Spillman, 2014; Kasper et al., 2019). This study examines the risk of moving to a residential care setting with and without nursing care based on older adults’ demographic and health characteristics, as well as the composition, size, shared task, and role type of their care networks (i.e., multiple persons offering support).

## Older Adult Characteristics Predicting a Transition to Residential Care

It is well established that the decision to transition to residential care, encompassing settings like assisted living and others that offer communal living arrangements, meals, and multiple levels of assistance with daily activities, is shaped by various characteristics of the older adult. Key sociodemographic factors like being older, female, non-Hispanic White, or having low educational attainment, along with health factors like dementia, multimorbidity, and functional disability, are common predictors of nursing home admission (Berete et al., 2022; Cepoiu-Martin et al., 2016; Carter et al., 2021; Gaugler et al., 2009; Jørgensen et al., 2019; Luppa et al., 2010; Wolf et al., 2018). However, fewer studies have examined how older adults’ personal characteristics predict relocation to residential care settings other than nursing homes. One study determined that “elder orphans,” or socially and physically isolated older adults without caregiving support, have a significantly higher risk of moving to residential care compared to both non- or at-risk elder orphans (Roofeh et al., 2022). In addition, Chyr et al. (2020) observed that incident transition to residential care settings among community-dwelling older adults was more common than transitioning to nursing homes from 2011 to 2018. Han and colleagues (2017) also revealed that assisted living facilities often care for and admit residents with significant care needs, including some comparable to those seen in nursing homes, despite having fewer staff resources. Research indicates a decline in nursing home admissions and growth in other forms of residential care (Han et al., 2017); therefore, it is important to consider various types of residential care settings.

## Caregiver Involvement Predicting a Residential Care Transition

Among older adults who receive help, including those with dementia or those on the verge of admission to a nursing home, a vast majority rely on family or unpaid caregivers (Hainstock et al., 2017; Kasper et al., 2015; Ydstebø et al., 2020). Indeed, previous research has considered the role caregivers play in nursing home admission (Buhr et al., 2006; Jørgensen et al., 2019; McClendon et al., 2006; Spillman & Long, 2009; Wolff et al., 2018; Yaffe et al., 2002). Most of these studies focus on one primary caregiver (Wolff et al., 2018) or caregiver stress and burden (Spillman & Long, 2009; Yaffe et al., 2002) as predictors of nursing home admission, largely excluding alternative residential settings.

## Care Network Characteristics and Guiding Theoretical Framework

There remains a significant gap in understanding the implications of the care *network* characteristics—specifically, factors related to the broader helper network or multiple persons providing support—on residential care admission. With some exceptions (e.g., Andersson & Monin, 2018; Fast et al., 2004; Hu et al., 2023; Koehly et al., 2015; Leggett et al., 2023; Spillman et al., 2020; Xu et al., 2021), empirical caregiving research has traditionally focused on a primary caregiver or caregiver–recipient dyad, although having multiple caregivers involved is common, particularly among racial/ethnic minorities and those with greater care needs like dementia (Freedman & Spillman, 2014; Hu et al., 2023).

Care networks could have dual effects on care outcomes, like residential care admission. On one hand, a shared care network, as opposed to a primary care network, could offer benefits (Hu et al., 2023), including distributed responsibilities, diverse skill sets, emotional support, and increased vigilance. These factors can mitigate the adverse effects of unmet needs, benefiting both the care recipient and network members and potentially reducing the risk of residential care admission. On the other hand, multiple caregivers may introduce challenges such as coordination difficulties, conflicting approaches, and caregiver burden, particularly in complex care scenarios like dementia (Spillman et al., 2020). Altogether these challenges could increase the likelihood of residential care admission. Understanding care network dynamics is necessary for developing effective interventions and policies that promote aging in place.

We situate this study in the “Framework to Assess the Changing Demography of Late-Life Family Caregiving” by Freedman and colleagues (2024), which contextualizes family caregiving within the broader landscape of older adults’ care needs, available alternatives, and resulting outcomes. Emphasizing the significance of care networks over individual caregivers, this framework acknowledges the evolving nature of networks influenced by demographic, health-related, or socioenvironmental factors. Our focus lies specifically in exploring the linkages among the older adults’ care needs, their potential and actual care network, and alternative supports (i.e., residential care). Drawing from prior studies, we focus on network size, relationship composition, care domain task sharing, and the number of in-network specialists and generalists (Leggett et al., 2023; Spillman et al., 2020) to characterize older adult care needs and care network.

Regarding network size, the literature presents mixed findings. Certain studies suggest that having fewer caregivers may be advantageous. For example, Gaugler (2009) noted that having any caregiver increased the risk of moving to a nursing home, and Jordan et al. (2024) acknowledged a care paradox in which self-directing one’s own care was associated with a lower risk of institutionalization or death compared with having a spouse or adult caregiver or a paid employee helper. Distress and challenges in coordinating care could arise with multiple caregivers, consequently leading to poor outcomes for all involved (Xu et al., 2021). Conversely, others point to the benefits of larger networks, emphasizing the strength in numbers. For instance, Carter et al. (2021) found that individuals with dementia using paid support services but receiving fewer family care hours had high rates of admission to long-stay residential care, implying the significant impact of family care provision. Larger care networks may enable more collaboration (Ellis et al., 2023), reducing the responsibilities of the care recipient or primary caregiver.

In terms of relationship composition, spouses and children tend to emerge as primary sources of assistance to older adults (Freedman et al., 2024). Cantor’s hierarchical-compensatory model (1979) posits that paid support or nonimmediate family support is sought when immediate family is lacking or when it complements and supplements family care. Yet, the focus is on when caregivers are added, not necessarily when they relinquish their roles. Nevertheless, extended family members and nonfamily are also known to play significant roles in care provision (Fast et al., 2004; Freedman & Spillman, 2014). Thus, understanding *who* is in these care networks is important to consider prior to residential care admission.

The literature on task-sharing of care activity domains, such as medical, household, mobility, self-care, and transportation, across network members is limited but growing (Ellis et al., 2023; Leggett et al., 2023; Litwak, 1985; Spillman et al., 2020). Hu and colleagues (2023) uncovered that care recipients within a shared care network (i.e., a network that is sharing care tasks) were less likely to have unmet needs compared to those with similar-sized primary care networks. Hence, networks that are more collaborative may reduce the risk of a residential care transition. Litwak’s (1985) task-specific model highlights the varied functions among network members who provide specialized types of support based on their emotional connection, geographic proximity, and level of commitment to the care recipient. Taking a similar approach as Spillman et al. (2020), we distinguish between caregivers acting as specialists, aiding in a single activity domain, and generalists, assisting across multiple domains. While collaborative task sharing could be helpful, having more generalists in one’s network may also signify overloaded networks.

## Current Study

The decision or necessity to transition from community-based living to residential care is a complicated process influenced by various personal demographics and care network factors. Limited research has focused on the confluent role of care *networks* instead of a sole, primary caregiver on residential care admission. This study aims to address this gap by employing competing risk analysis, as opposed to traditional survival analyses that may fail to account for the competing risk of death, to assess residential care admission (Carter et al., 2021; Roofeh et al., 2022). Using data from the National Health and Aging Trends Study (NHATS) Rounds 1–9, we advance this area of research by exploring the characteristics of care networks that protect or add risk to older adults’ hazard of transitioning to any residential care setting like assisted living or a nursing home. Given the limited and mixed findings on networks of care, we hypothesize that greater complexity or involvement in care provision among networks—such as larger size, inclusion of distant or nonfamily members, and shared assistance across multiple domains, particularly medical care—predicts a greater risk of relocation.

## Method

### Data and Analytic Samples

NHATS is a panel study of Medicare beneficiaries ages 65 and older living in the contiguous United States starting in 2011 (Montaquila et al., 2012). NHATS conducts annual interviews. During these interviews, older adult respondents were queried to create a comprehensive roster of unique affiliated individuals, including spouses/partners, children, all household members, emergency contacts, persons cared for by the respondent, and up to five close confidantes. The roster details each person’s relationship to the older adult, whether they provided assistance, and the specific activities they helped with (i.e., mobility, self-care, household tasks, medical care, and/or transportation) in each round (Freedman et al., 2023). We use this information to derive measures of the full care network for each NHATS respondent in each round.

In 2011, there was a total of 8,245 respondents. We excluded those not living in the community (*n *= 411), missing education (*n* = 82), and without a network member identified (*n* = 667) from Round 1. Thus, the initial analytic sample for this study was *N* = 7,085. Only the original cohort from 2011 was followed until 2019.

### Outcome: Relocation from Community to Residential Care

The primary outcome under investigation was the relocation to residential care subsequent to participation in NHATS 2011 as a community-dwelling resident. Residential care settings are broadly defined in NHATS and include free-standing nursing homes, assisted living (or personal care or group homes) facilities, independent living facilities, continuing care communities, and other retirement or senior housing facilities. Places that offer multiple levels of care, provide meals, or provide assistance with daily activities are eligible for a facility questionnaire, which determines both the type of place and level of care received by the NHATS participant (Freedman et al., 2023). Similar to Roofeh et al. (2022), short-term postacute stays in nursing homes are not considered relocation; instead, we follow NHATS’ convention of including only individuals who move to a nursing home setting who are not expected to return home. Participants were followed until residential care relocation, death, or end of the study period (2019—before the COVID-19 pandemic). Only the initial admission to residential care was taken into account in the analysis.

### Care Network Predictors

We examined several factors related to care networks. First, we considered the *size* of the full care network determined by the count of all unique affiliated individuals mentioned during the sample person’s interview.

Additionally, we included the *type of relationship* to the older adult participant available in the network. Specifically, we assessed if the full care network included a spouse or partner, daughter or daughter-in-law, son or son-in-law, nonimmediate family (e.g., cousins, aunts/uncles), and nonfamily (e.g., friends, neighbors, etc.).

To establish *shared tasks* among network members, we first identified four domains: medical tasks, such as attending medical visits and keeping track of medications; household duties, including laundry, shopping, meal preparation, bill payment, and banking; mobility or self-care activities like moving around indoors or outdoors, eating, bathing, toileting, and dressing; and transportation needs involving going places outside of the home. It should be acknowledged that care network helpers are included no matter the reason, whether they provided help for health and functioning (i.e., caregivers who are eligible for the National Study of Caregivers [NSOC]) or other reasons (e.g., spouse helped with hot meals or laundry because that is how tasks are divided in their household). We broaden this definition to all helpers because not all “caregiving” per se is related to health and functioning. Quantifying task sharing involved determining the number of helpers providing assistance in each domain, with a threshold applied: if the sum of helpers was less than two, it indicated no task sharing; if two or more, task sharing was present.

We also constructed the classification of helpers as either *specialists* (network members assisting with one activity domain) or *generalists* (assisting with multiple domains), as defined by Spillman and colleagues (2020). The analysis considered the count of generalists and specialists. For each of these counts of generalists, we constructed three-level indicators: no generalist, one generalist, and more than one generalist. The categorization for specialists followed the same approach.

### Older Adult Characteristics

Sociodemographic characteristics of older adults included age (continuous), gender (0 = *female*, 1 = *male*), and education level (0 = *high school or less*, 1 = *more than high school*). Race/ethnicity was grouped as non-Hispanic (NH) White, NH Black, and Hispanic/NH Others, which included American Indian, Alaska Native, Asian, and Pacific Islander. All demographic factors were obtained from the Round 1 (2011) NHATS interview.

Health factors, such as comorbidity and dementia status, of the older adults were also considered. Participants were asked about their history of major chronic diseases (e.g., heart disease, diabetes), where each disease was recorded as a dichotomous variable (1 = *yes*, 0 = *no*). A composite variable was created by summing these disease variables (ranging from 0 to 9). Dementia was removed from the chronic condition list as the dementia classification was utilized. Classification of dementia in NHATS is based on three main sources: self- or proxy-reported diagnoses from physicians, scores from the AD8 Dementia Screening Interview given to proxies, and cognitive test outcomes. These tests evaluate memory, orientation, and executive function. In cases where proxies are involved and cognitive tests are incomplete, diagnosis reports and AD8 scores are considered. NHATS categorizes outcomes into *no dementia*, *possible dementia*, and *probable dementia*, representing different levels of cognitive impairment. Details of this classification are outlined in a technical paper by Kasper et al. (2013).

Marital status, living arrangement, and functional status were not included, as they are indirectly assessed through the care network variables (e.g., spouse in-network, help with self-care/mobility, etc.).

### Statistical Analysis

Data were weighted using Wave 1 sampling weights to account for nonresponse bias (Montaquila et al., 2012), and all analyses were conducted using Stata version 17 (Stata Corp., College Station, TX). This study employed competing risk survival regressions using Fine and Gray models (1999) to investigate the transition from community-based living to residential care, with death as a competing risk. The competing event, death, impedes the occurrence of the event of interest, residential care. Censoring occurred when individuals did not experience the event of interest (i.e., transition to residential care or death) during the study period but were no longer followed for various reasons, such as loss to follow-up or the end of the observation period. These individuals were still included in the analysis up to the point at which they were censored, meaning their data were used until the time they were no longer being followed. Attrition accounted for *n* = 1,468 participants (20.72%). By the end of the study period (i.e., 2019), 2,158 participants (30.5%) of the initial analytical sample (*N *= 7,085) still remained in the community.

We considered sociodemographic factors of the older adult as well as the characteristics of their care networks. The base model (Model 1) includes the care recipient characteristics alone. Subsequential models add the following: network size (Model 2), Model 3 (relationship composition), Model 4 (task-sharing), and Model 5 (number of generalists/specialists). These results are presented using subdistribution hazard ratios (SHR) and 95% confidence intervals (CI).

In sensitivity analyses, we included time-varying covariates to adjust for changes in older adult age and health and care network characteristics over time. Additionally, we conducted separate Cox hazard survival analysis models while applying Round 1 cohort weights to account for the complex survey design (Freedman et al., 2020) for residential care and nursing homes without considering the competing risk of mortality. Last, we modified the inclusion criteria to encompass solely caregivers offering assistance for health and functioning purposes instead of the broader helper or potential care network.

## Results

### Descriptive Statistics

Table 1 reports descriptive statistics at baseline for the 2011 NHATS sample. Based on the weighted sample, participants were 75 years on average, with 55.6% being female. The racial–ethnic composition included 80.9% NH White, 8.35% NH Black, and 10.7% classified as Hispanic/NH Others. Approximately half of the participants had education beyond high school (50.8%). On average, participants had between two and three (*M* = 2.4, range = 0–9) chronic conditions. About one in five (19.3%) participants had either possible or probable dementia.

**Table 1.** Baseline Characteristics (*N* = 7,085)

Variables	Weighted sample		Unweighted sample			
	*M* or %	95% CI	*M* or %	*SD*	Min	Max
Older adult characteristics						
Age (years), *M*	74.8	[74.6–75.0]	77.3	7.7	65	105
Gender						
Female	55.6	[54.1–57.1]	57.5			
Male	44.3	[42.9–45.9]	43.5			
Race/ethnicity						
Non-Hispanic White	80.9	[79.1–82.6]	68.2			
Non-Hispanic Black	8.4	[7.6–.9.2]	22.4			
Non-Hispanic other/Hispanic	10.7	[9.3–12.3]	9.4			
Education						
High school or less	49.3	[46.9–51.6]	54.8			
More than high school	50.8	[48.4–53.1]	45.2			
Chronic conditions	2.4	[2.4–2.5]	2.6	1.6	0	8
Dementia classification						
Probable dementia	8.6	[8.0–9.6]	12.5			
Possible dementia	10.6	[9.3–12.0]	12.8			
No dementia	80.6	[79.0–82.4]	74.7			
Care network characteristics						
Presence of specific relationship						
Network size, *M*	5.0	[4.9–5.1]	5.1	2.4	1	21
Spouse in-network	59.3	[57.9–60.7]	51.9			
At least one daughter or daughter-in-law	73.6	[72.0–74.9]	75.3			
At least one son or son-in-law	73.7	[72.4–74.9]	73.78			
At least one nonimmediate family	7.5	[6.9–08.2]	9.0			
At least one nonfamily	41.1	[39.6–42.6]	41.7			
Shared network tasks						
Medical	5.7	[5.1–6.3]	7.7			
Household	15.3	[14.2–16.4]	19.1			
Mobility & self-care	6.4	[5.8–7.0]	9.4			
Transportation	19.4	[18.1–21.0]	21.7			
Type of helpers						
Number of generalists						
0	51.6	[50.2–53.0]	47.9			
1	38.6	[37.1–40.1]	39.0			
2+	9.8	[9.0–10.7]	13.1			
Number of specialists						
0	44.5	[43.2–45.9]	44.1			
1	41.8	[40.5–42.8]	39.9			
2+	13.8	[12.9–14.8]	16.0			

*Notes*: CI = confidence interval. Round 1: full sample weights were applied.

Individuals had an average of five people in their care network, though this ranged from 1 to 21 network members identified. In terms of composition, 59.3% had a spouse, 73.6% had at least one daughter or daughter-in-law, 73.7% had at least one son or son-in-law, 7.5% had at least one nonimmediate family, and 41.1% had at least one nonfamily person in their network. Additionally, 48.4% had at least one specialist, and 55.5% had at least one generalist.

The initial model incorporated characteristics of older adults. Advanced age (SHR = 1.09, 95% CI: 1.08–1.11, *p* < .001), higher educational attainment (SHR = 1.20, 95% CI: 1.09–1.32, *p* < .000), and a greater number of chronic conditions (SHR = 1.30, 95% CI: 1.23–1.37, *p* < .001) were identified as factors increasing the likelihood of transitioning to residential care. Individuals with probable dementia faced a 2.62 times greater risk (SHR = 2.62, 95% CI: 1.99–3.44, *p* < .000), while those with possible dementia faced a 1.64 times greater risk (SHR = 1.64, 95% CI: 1.26–2.15, *p* < .001) compared to those without dementia. Conversely, being male (SHR = 0.74, 95% CI: 0.61–0.90, *p* = .002), NH Black (SHR = 0.60, 95% CI: 0.46–0.76, *p* < .001), and individuals of Hispanic/NH other backgrounds (SHR = 0.47, 95% CI: 0.31–0.72, *p* < .001) were associated with a decreased likelihood of transitioning out of the community. Models 2 through 5 incorporate sociodemographic characteristics of older adults and sequentially introduce care network factors (see Table 2).

**Table 2.** Older Adult and Care Network Characteristic Risks for Residential Care Relocation With Death as a Competing Risk

Variables	Model 1	Model 2	Model 3	Model 4	Model 5
	SHR [95% CI]	SHR [95% CI]	SHR [95% CI]	SHR [95% CI]	SHR [95% CI]
Older adult characteristics					
Age	1.09 [1.08–1.11]^***^	1.10 [1.08–1.11] ^***^	1.08 [1.07–1.10] ^***^	1.07 [1.05–1.08] ^***^	1.07 [1.06–1.08] ^***^
Gender (male)	0.74 [0.61–0.90] ^**^	0.79 [0.65–0.96] ^*^	0.93 [0.75–1.15]	0.75 [0.61–0.92] ^**^	0.82 [0.67–1.00] ^*^
Education (more than HS)	1.20 [1.09–1.32] ^***^	1.18 [1.07–1.29] ^**^	1.18 [1.08–1.30] ^***^	1.20 [1.10–1.32] ^***^	1.21 [1.11–1.33] ^***^
Race/ethnicity (ref: non-Hispanic White)					
Non-Hispanic Black	0.60 [0.46–0.76] ^***^	0.53 [0.41–0.69] ^***^	0.54 [0.42–0.69] ^***^	0.50 [0.39–0.64] ^***^	0.55 [0.43–0.71] ^***^
Non-Hispanic Other/Hispanic	0.47 [0.31–0.72] ^***^	0.44 [0.29–0.67] ^***^	0.49 [0.32–0.75] ^**^	0.40 [0.26–0.62] ^***^	0.44 [0.29–0.67] ^***^
Chronic conditions	1.30 [1.23–1.37] ^***^	1.26 [1.2–1.33] ^***^	1.32 [1.25–1.38] ^***^	1.02 [0.95–1.09]	1.06 [0.99–1.13]
Dementia (ref: no dementia)					
Probable dementia	2.62 [1.99–3.44] ^***^	2.68 [2.03–3.54] ^***^	2.69 [2.04–3.54] ^***^	1.71 [1.31–2.24] ^***^	1.99 [1.54–2.58] ^***^
Possible dementia	1.64 [1.26–2.15] ^***^	1.71 [1.31–2.24] ^***^	1.73 [1.33–2.29] ^***^	1.23 [0.93–1.62]	1.37 [1.05–1.80] ^*^
Care network characteristics					
Network size		1.09 [1.07–1.12] ^***^			
Presence of specific relationship					
Spouse in-network			0.49 [0.38–0.63] ^***^		
At least one daughter or daughter-in-law			0.57 [0.46–0.69] ^***^		
At least one son or son-in-law			0.66 [0.54–0.80] ^***^		
At least one nonimmediate family			0.71 [0.55–0.93] ^*^		
At least one nonfamily			1.83 [1.39–2.40] ^***^		
Shared network tasks					
Shared medical help				7.28 [5.76–9.21] ^***^	
Shared household help				3.10 [2.49–3.87] ^***^	
Shared mobility & self-care help				0.67 [0.54–0.84] ^***^	
Shared transportation help				0.57 [0.47–0.70] ^***^	
Type of helpers					
Number of generalists (ref: 0)					
1					2.08 [1.54–2.80] ^***^
2+					7.43 [5.61–9.84] ^***^
Number of specialists (ref: 0)					
1					0.96 [0.77–1.20]
2+					1.26 [0.99–1.62]

*Notes*: CI = confidence interval; Ref = reference category; SRH = subdistribution hazard ratio. Round 1: sampling analytic weights were applied. Model 1: older adult characteristics. Model 2: older adult characteristics + network size. Model 3: older adult characteristics + specific relationship. Model 4: older adult characteristics + shared tasks. Model 5: older adult characteristics + number of generalists & specialists. Obs. = 37,207.

^*^ *p* < .05; **p < .01; ***p < .001.

### Care Network Factors and Residential Care Relocation

#### Size

In Model 2, network size was introduced as an additional factor, revealing that a larger network size was associated with a 9% greater hazard of transitioning to residential care (SHR = 1.09, 95% CI: 1.07–1.12, *p* < .001). The direction and significance of the control variables remained the same, though there were minor fluctuations in subhazard ratios.

#### Composition

Subsequently, Model 3 integrated specific relationships within the care network. The presence of any family member in the network was associated with reduced risks of relocating to residential care. Specifically, the level of closeness played a significant role, with the following ranking from least to greatest risk: spouse (SHR = 0.49, 95% CI: 0.38–0.63, *p* < .001), daughter or daughter-in-law (SHR = 0.57, 95% CI: 0.46–0.70, *p* < .000), son or son-in-law (SHR = 0.66, 95% CI: 0.55–0.80, *p* < .001), and nonimmediate family (SHR = 0.80, 95% CI: 0.63–1.03, *p* = .011). In contrast, the inclusion of nonfamily members such as friends or neighbors was associated with an increased risk of relocation (SHR = 1.83, 95% CI: 1.39–2.40, *p* < .001). Gender was found to be nonsignificant in this model.

#### Task sharing

Model 4 explored shared tasks within the network, indicating that shared medical help (SHR = 7.28, 95% CI: 5.76–9.21, *p* < .001) was associated with a substantially increased risk of transitioning to residential care. Sharing household tasks also was associated with an increased hazard of moving (SHR = 3.10, 95% CI: 2.49–3.87, *p* < .001). Interestingly, shared mobility and self-care help (SHR = 0.67, 95% CI: 0.54–0.84, *p* < .001) and shared transportation help (SHR = 0.57, 95% CI: 0.47–0.70, *p* < .001) were associated with a lower hazard of moving. Chronic conditions and possible dementia were no longer statistically significant predictors once shared tasks were added to the model. Although the overall directions remained consistent, all controls exhibited reduced hazards with the exception of education and gender.

#### Number of specialists or generalists

In Model 5, the number of specialists and generalists were included. Having specialists was not significantly related to the risks of entering residential care (one specialist: SHR = 0.96, 95% CI: 0.77–1.20, *p* = .740; two or more specialists: SHR = 1.26, 95% CI: 0.99–1.62, *p* = .064). Having one generalist was associated with an increase in the hazard (SHR = 2.08, 95% CI: 1.54–2.80, *p* < .001), compared to having no generalists. Moreover, having two or more generalists facilitated an even greater incidence of relocation (SHR = 7.43, 95% CI: 5.61–9.84, *p* < .001).

### Sensitivity Analyses

The findings of the competing risk analysis with time-varying covariates had consistent directional trends as before, although there were reductions in the coefficient magnitudes for the time-varying covariates. One notable difference from the analysis without time-varying covariates emerged: the presence of two or more specialists significantly increased the risk of relocation by seven percent (SHR = 1.07, 95% CI: 1.02–1.12, *p* < .05).

We also conducted survival analyses without the competing event of death to test the robustness of the results. Although the direction and coefficient values align similarly with the competing risk analysis results, some distinctions were found. Specifically, network characteristics such as nonimmediate family shared transportation tasks, and having two or more specialists achieved significance in this context. Given the wide variability in care levels within residential care settings, ranging from independent living to skilled nursing, we conducted an additional survival analysis specifically focusing on the risk of nursing home admission. In comparison to the analysis encompassing various residential care types, neither education nor NH Black reached significance in any model. Also, the risk ratios for probable and possible dementia-predicting nursing home admission alone were substantially higher. All network characteristics were significant. Contrasting with residential care as the outcome, having mobility or self-care assistance increased the risk of nursing home relocation, while the presence of any number of specialists decreased it. Interestingly, the presence of one generalist in the network was associated with a lower nursing home relocation risk, but having two or more was associated with an increased risk.

To discern potential or available help and actual “caregiver” network members (Freedman et al., 2024; Wyman et al., 2023), we conducted analyses using models that included the additional inclusion criteria of being NSOC-eligible or providing care for health and functioning reasons. The results closely paralleled those obtained from the broader care network, exhibiting similar trends and statistical significance.

## Discussion

We aimed to add to the body of literature by investigating how broader care network characteristics (i.e., size, relationship composition, task sharing, and collaborator type) contribute to older adult care transitions. Given recent shifts away from traditional nursing homes and toward alternative housing and care options (De Boer et al., 2018), we considered residential care facilities with and without nursing care over a 9-year period. In addition, we factor in death as a competing risk. Similar to prior research predicting skilled nursing facility admissions, the results suggest that older adults’ demographic characteristics, explicitly being older, female, NH White, and health factors, having dementia or more chronic conditions, significantly influence the risk of moving to a residential care setting (Berete et al., 2022; Chyr et al., 2020; Jørgensen et al., 2019). Our contribution to previous literature is as follows: Individuals with large networks in which helpers take on multiple areas of assistance without close family helpers were at higher risk for relocating.

### Care Network Factors and Residential Care Relocation

In general, we interpret our results to indicate that older adults with large or complex networks were at greater risk for residential care moves. Although the adage “many hands make light work” is commonly embraced, this study indicates that an abundance of caregivers could present challenges, perhaps better resonating with the notion of “too many cooks in the kitchen.” As a team of caregivers, conflicting opinions or logistical hurdles may arise (Doekhie et al., 2018), emphasizing the need for strategies such as co-management, focused communication, and clarified responsibilities to address these challenges effectively. However, we do not advocate for reducing available structural support; rather, we stress that the timing, quality, and coordination of assistance are crucial alongside the maintenance of robust care networks. In this context, an external professional can play a vital role in identifying potential issues, providing guidance on problem management, and facilitating decision making.

Additionally, it is possible these networks form in response to increased care needs (Freedman et al., 2024), as evidenced by the impact of shared medical tasks and dementia. Another study similarly found that while older adults with multiple chronic conditions and dementia have larger care networks compared to those without such health concerns, they face greater challenges in meeting their care needs (Beach et al., 2020). Therefore, prioritizing policies that ensure equitable and timely access to home and community-based services (e.g., respite, adult day care programs, home health, caregiver training opportunities) as well as compensation mechanisms for family caregivers, such as direct financial stipends or tax credits, is critical. Such measures could alleviate financial strain, bolster caregivers’ commitment and confidence, and improve care quality. This support is essential for balancing caregiving responsibilities with other aspects of life, potentially reducing premature transitions to residential care settings.

The presence of a spouse within the care network was associated with the lowest risk of relocation—followed by daughters, then sons, then nonimmediate family—a finding consistent with prior research predicting nursing home admission (Jordan et al., 2024; Jørgensen et al., 2019). In line with Cantor’s hierarchical-compensatory model (1979), the significance of spouses and adult children in contrast to the nonsignificant role of nonimmediate family members suggests that individuals primarily rely on their spouse or adult child for support and then turn to distant family, friends, or neighbors. Additionally, even more distant family relatives, such as a grandchild or cousin, could offer significant benefits to the ability to remain in the community. Having nonfamily in their care network was linked to a higher likelihood of moving. To illustrate, a widow who lives alone and lacks nearby family may turn to a neighbor, friend, or paid helper. Although these individuals can offer valuable assistance, they may serve as a secondary or temporary solution that may not meet long-term care needs effectively.

These findings suggest that caregiver network availability and involvement are paramount to older adults’ ability to age in place, which is the most desired outcome for many older adults (AARP, 2021). At the policy level, insights from this research can guide decisions on when, where, and how to supplement aging adults with additional support. For example, policies could focus on strengthening existing care networks or enhancing social support systems specifically lacking a spouse, adult child, or other close relative caregiver (Tang & Lee, 2011). Regular and proactive assessment of an individual’s care network can reveal gaps in care coverage, allowing for intervention before “breaking points” related to safety, physical dependency, or loss of a caregiver (Auriemma et al., 2024; Lam et al., 2023). Targeted aging-in-place interventions could include strengthening knowledge of home- and community-based services, peer support initiatives, or in-home monitoring technology that address the unique challenges faced by those lacking traditional caregiving arrangements (Auriemma et al., 2024; Gettel et al., 2021; Tang & Lee, 2011). For example, the Community Aging in Place Advancing Better Living for Elders program, which combines nursing, occupational therapy, and home modification services, has been effective in reducing disability and enhancing the independence of older adults, thereby easing the burden on caregiving networks that may be stretched thin (Fields et al., 2022). Overall, the findings further substantiate existing literature indicating that family caregivers are a central component of aging in place (Lam et al., 2023), but when these key helpers are limited or absent, well-planned supplementary support is necessary to maintaining independence and quality of life.

When more medical or house-related tasks were collaboratively undertaken, the incidence of residential relocation increased. This may suggest that these particular care needs exceed the helper network’s resources and capacities. However, sharing mobility and self-care or transportation tasks amongst network caregivers did protect against a move, suggesting that backup for these critical activities may be important. Mobility and self-care may most likely be carried out by a spouse or child, reducing the odds of a transition (Ali et al., 2022). Individuals may view self-care as more personal and desirable to manage at home. Conversely, medical care needs may be seen as better suited for specialized facilities, potentially beyond caregivers’ comfort level or expertise. This emphasizes the necessity for specialized training and support programs or interventions tailored to caregivers’ roles and challenges, ensuring they are adequately prepared to handle diverse care needs. In cases of particularly complex tasks, it may be advantageous to share the responsibilities with a professional caregiver (e.g., home health aide) to enable the ability to age in place, provided these supports are accessible and affordable.

The insignificant role of transportation task sharing in influencing a decision to move may be attributed to its sporadic and unplanned nature, as well as its lower intensity compared to other caregiving activities (Spillman et al., 2020). Also, its impact may vary depending on the composition of the network. For instance, providing transportation assistance may not be burdensome for a retired spouse or someone with a flexible job. Whereas, if multiple children are available to take turns driving to doctor’s appointments, the responsibility does not fall solely on one individual. These results may also reflect the inclusion of care settings besides skilled nursing facilities like independent living. Nonetheless, accessible transportation services remain essential, allowing caregivers to dedicate more time and energy to other important care-related needs.

Having generalists, referring to helpers engaged in multiple domains of care assistance, was found to be associated with a higher likelihood of a move, whereas specialists, focusing on a single type of care task, did not significantly predict relocation. The risk of relocation was further heightened in networks with more than one generalist. In other words, generalists may face challenges managing multiple tasks, potentially linked to complex care needs or caregiver burden. Conversely, focusing on one task may be more manageable. Marcum and colleagues (2020) explored perceived primary caregiver roles within networks of multiple caregivers and proposed that a lack of consensus could potentially result in interpersonal conflict among members. While primary caregivers and generalists are not necessarily synonymous, these findings could also extend to generalists. As highlighted by Spillman et al. (2020), there is a compelling case for caregiver training on task delegation, time management, and help-seeking to better handle multiple responsibilities without becoming overwhelmed. Additionally, it could be valuable to identify caregivers at risk for role-related burnout such as generalists, or those with close proximity and/or fewer resources (Ellis et al., 2023).

### Limitations and Future Directions

Several limitations should be acknowledged. First, we grouped all residential care settings together (including nursing homes), encompassing housing options with diverse amenities, care provisions, and admission policies. Our sensitivity analysis highlighted emphasized risks associated with dementia when multiple members share increased care needs (e.g., self-care and mobility tasks), yet having one generalist or specialist may offer protection for nursing home admission. These trends were not as evident for admission to all types of residential care. Thus, researchers should exercise caution in generalizing findings to specific care settings, as the outcomes may vary significantly depending on the composition and dynamics of the care environment. In addition, we assessed the first permanent moves out of the community rather than acute stays or multiple care transitions (e.g., moving through a continuing care continuum) (Hainstock et al., 2017). Furthermore, our analysis is based on data collected prior to the COVID-19 pandemic. Examining changes pre- and post-COVID-19, along with utilizing more recent data, would be fruitful.

This study also lacks insight into the intricate behind-the-scenes decision-making of older adults or caregivers that led to the voluntary or involuntary move to a residential facility (Afram et al., 2015; Kasper et al., 2019; Vandepitte et al., 2018). While we applied primary sampling weights, our analysis was constrained by the inability to incorporate stratification and clustering variables in the competing risk analysis using Stata’s “stcrreg” command. Lastly, the use of the initial cohort weight (e.g., 2011 for the 2011 cohort) does not explicitly consider loss to follow-up that may occur in subsequent rounds (Freedman et al., 2020). These limitations may have impacted the generalizability of our findings.

A potential valuable future direction for research would be examining how paid help or services (e.g., medical providers, home health aides, community services) interact with family care networks. Additionally, future research should also assess how family care networks structurally and functionally evolve after a major life event (e.g., care recipient dementia diagnosis) or once the older adult moves to a care facility. As Gaugler and Mitchell (2022) noted, family involvement does not end with postrelocation. More details about the care network, such as geographical distance from the care recipient, family or work-related demands, subjective discord or appraisals amongst network members, and emotional support provided as a care activity domain, warrant further attention. Other unexplored factors, such as aspects related to the home or neighborhood environment and accessibility, may contribute to the complexity of a residential care move, as well (Granborn et al., 2019). New insights on care network dynamics and heterogeneity may hold implications for the design or advancement of interventions and policies that more effectively support the unique health and well-being needs of individuals aging in place and all caregivers involved.

### Conclusion

This study illuminates the combined impact of individual and care network factors on the risk of relocating to residential care. By pinpointing high-risk individuals based on the strength and composition of their care networks, the study offers valuable insights for future aging-in-place interventions aimed at preventing or delaying such transitions. Specifically, interventions should prioritize individuals lacking a partner or daughter in their network, as well as those requiring medical or household support, shared among multiple caregivers, particularly for those with dementia or disabilities. Although larger networks may initially appear advantageous, they may also lead to issues, such as a lack of coordinated care and stretched resources for generalists. Recognizing the availability and challenges within care networks can be transformative in shaping policies and practices related to additional support and resources for aging in place. Given the likelihood of care transitions for many older adults, providing guidance counseling and support to care networks during these periods is crucial. Ultimately, enhancing care management throughout networks may alleviate burden, enhance care quality, and prolong older adults’ independence.

## Data Availability

Data are publicly available at nhats.org. The research was not preregistered.
